# Type 1 Innate Lymphoid Cells Are Proinflammatory Effector Cells in Ischemia-Reperfusion Injury of Steatotic Livers

**DOI:** 10.3389/fimmu.2022.899525

**Published:** 2022-06-27

**Authors:** Jiman Kang, Jedson R. Liggett, Digvijay Patil, Suman Ranjit, Katrina Loh, Anju Duttargi, Yuki Cui, Kesha Oza, Brett S. Frank, DongHyang Kwon, Bhaskar Kallakury, Simon C. Robson, Thomas M. Fishbein, Wanxing Cui, Khalid Khan, Alexander Kroemer

**Affiliations:** ^1^ MedStar Georgetown Transplant Institute, MedStar Georgetown University Hospital and the Center for Translational Transplant Medicine, Georgetown University Medical Center, Washington, DC, United States; ^2^ Department of Biochemistry and Molecular & Cellular Biology, Georgetown University, Washington, DC, United States; ^3^ Naval Medical Center Portsmouth, Portsmouth, VA, United States; ^4^ Department of Oncology, Lombardi Comprehensive Cancer Center, Georgetown University Medical Center, Washington, DC, United States; ^5^ Department of Pathology, MedStar Georgetown University Hospital, Washington, DC, United States; ^6^ Departments of Anesthesiology and Medicine, Beth Israel Deaconess Medical Center, Harvard Medical School, Boston, MA, United States

**Keywords:** Type 1 innate lymphoid cells, T-bet, ischemia-reperfusion injury, fatty liver disease, liver transplantation, phasor fluorescence lifetime imaging, innate lymphoid cells, natural killer cells

## Abstract

Innate lymphoid cells (ILCs), the most recently described family of lymphoid cells, play fundamental roles in tissue homeostasis through the production of key cytokine. Group 1 ILCs, comprised of conventional natural killer cells (cNKs) and type 1 ILCs (ILC1s), have been implicated in regulating immune-mediated inflammatory diseases. However, the role of ILC1s in nonalcoholic fatty liver disease (NAFLD) and ischemia-reperfusion injury (IRI) is unclear. Here, we investigated the role of ILC1 and cNK cells in a high-fat diet (HFD) murine model of partial warm IRI. We demonstrated that hepatic steatosis results in more severe IRI compared to non-steatotic livers. We further elicited that HFD-IRI mice show a significant increase in the ILC1 population, whereas the cNK population was unchanged. Since ILC1 and cNK are major sources of IFN-γ and TNF-α, we measured the level of *ex vivo* cytokine expression in normal diet (ND)-IRI and HFD-IRI conditions. We found that ILC1s in HFD-IRI mice produce significantly more IFN-γ and TNF-α when compared to ND-IRI. To further assess whether ILC1s are key proinflammatory effector cells in hepatic IRI of fatty livers, we studied both *Rag1* ^−/−^ mice, which possess cNK cells, and a substantial population of ILC1s versus the newly generated *Rag1* ^−/−^ *Tbx21* ^−/−^ double knockout (Rag1-Tbet DKO) mice, which lack type 1 ILCs, under HFD IRI conditions. Importantly, HFD Rag1-Tbet DKO mice showed significant protection from hepatic injury upon IRI when compared to *Rag1* ^−/−^ mice, suggesting that T-bet-expressing ILC1s play a role, at least in part, as proinflammatory effector cells in hepatic IRI under steatotic conditions.

## Introduction

Ischemia-reperfusion injury (IRI) is an unavoidable consequence of organ transplantation that can contribute to early allograft failure and increases the risk for subsequent allograft rejection (1). Hepatic steatosis has been identified as an independent risk factor for greater severity in ischemia-reperfusion injury (2). Unfortunately, given the increasing demand for liver transplantation and relative lack of available organs, marginal allografts, including those with hepatic steatosis, are being considered for use, given careful donor to recipient matching. This has led to significant research efforts to identify modifiable immune mediators to minimize IRI within these marginal organs.

Innate lymphoid cells (ILCs) are the most recently described family of lymphoid cells and are known to play fundamental roles in the first-line defense of epithelial barriers (3, 4), tissue homeostasis, and immune regulation through the activation of host-derived cytokine expression (5, 6). Specifically, Group 1 ILCs are a subset of ILCs that include Type 1 ILC (ILC1s) and conventional natural killer (cNK). Both ILC1s and cNK cells potently secrete TNF-α and IFN-γ (7) in a T-bet transcription factor-dependent manner; Eomesodermin (Eomes) is required only for the maturation of cNK, not ILC1 (8, 9). Moreover, T-bet expression is required for cNK cell maturation in stages, which are elicited by changes in the expression of CD27 and CD11b (8, 9). In this regard, CD11b^−^ CD27^+ ^ NK cells are the most immature NK cells, whereas mature CD11b^+ ^CD27^+ ^(double positive, DP) NK cells differentiate into terminally mature NK (CD11b^+^CD27^−^) cells. Further, while cNK cells are known to circulate throughout the body to eradicate deformed cells in a cytotoxic manner, ILC1s reside predominately within liver tissue (3, 6, 10) and elicit a variety of functions. It has previously been noted that ILC1s can serve a protective function through the production of IFN- γ, and upregulation of Bcl-xL, however, it is also known that these immune cells are prone to overactivation and dysregulation, contributing to autoimmune disorders (11). Additionally, recent work in hepatocellular carcinoma (HCC) using human tissue has associated ILC composition with HCC outcome, specifically denoting plasticity of cNK cells into tumor ILC1 (12).

While the activation of cNK cells has been associated with hepatic IRI (13), the role of ILC1s remains largely understudied. Given the resident and innate nature of ILC1s, the contribution to autoimmune disease, and plasticity of cNK cells to ILC1, we hypothesized that ILC1s are contributors to hepatic IRI, specifically in the setting of hepatic steatosis. In this present work, we utilized a high-fat diet (HFD) murine model of partial warm ischemia-reperfusion injury to elicit the presence of ILC1s in hepatic IRI. We then classified the function of ILC1 in comparison to cNK cells in IRI using *Rag1^−/−^* single knockout mice, which lack T and B cells but retain full ILC1 and cNK function versus *Rag1* ^−/−^ *Tbx21* ^−/−^ double knockout (Rag1-Tbet DKO) mice, which lack T and B cells as well as T-bet-dependent ILC1 cells but have retained Eomes^+^ cNK cells. Taken together, we report that ILC1s are a proinflammatory effector subset, driving IRI through the release of IFN-γ and TNF-α.

## Materials and Methods

### Mice

C57BL/6, *Rag1*^-/-^ and *Tbx21*^-/-^ (formal gene name *Tbx21*) mice were purchased from The Jackson Laboratory (Bar Harbor, ME) Rag1-Tbet DKO mice were obtained through crossbreeding of *Rag1^−/−^* mice with *Tbx21^−/−^* mice. Genotyping for Rag1-Tbet DKO was performed by Transnetyx. Mice were fed either standard chow (normal diet, ND) or a lard-based high fat diet (HFD). The ND, consisting of 4.09 kcal/gram,13.4% kJ/fat, was purchased from Lab Diet (St. Louis, MO). The HFD, consisting of 5.10 kcal/gram, 60% kJ/fat, was purchased from TestDiet (St. Louis, MO). ND was started at week four of life and maintained for 12-15 weeks for the ND group. For the HFD group experiment, HFD feeding was started between weeks five and seven of life. HFD continued for 12 weeks. All mice were bred and maintained within a pathogen-free facility in the Division of Comparative Medicine at Georgetown University Medical Center, with a standard 12-hour light-dark cycle. All procedures on animal subjects were fully approved by the Georgetown University Institutional Animal Care and Use Committee (protocol #2016-1351).

### Partial Warm Ischemia-Reperfusion Injury Model

Mice were anesthetized using 2% isoflurane and oxygen inhalation. After a midline laparotomy, an atraumatic micro clip was applied to the hepatic hilus. Mice were subjected to 45 min of partial warm hepatic ischemia, which closely corresponds to the average warm ischemia time during human liver transplantation at our institution and others. After 45 min of hepatic ischemia, the clip was removed to initiate liver reperfusion, and the peritoneum was reapproximated with sutures and skin closed with staples. After completion of the operation, the mice were returned to their cage. All analysis was performed after 24 hours of reperfusion. Whole blood was collected by direct cardiac puncture as a terminal procedure. The left lobe of the liver and the whole spleen were collected. Control mice were subjected to anesthesia with 2% isoflurane and oxygen inhalation and subjected to midline laparotomy, whole blood collection *via* cardiac puncture, and collection of the entire liver.

### Histology and Immunohistochemistry

Hematoxylin and eosin (H&E) staining was performed on five-micron sections from formalin-fixed paraffin-embedded liver tissues that were de-paraffinized with xylenes and rehydrated through a graded alcohol series. H&E staining was completed, followed by rehydration through a graded alcohol series using Autostainer XL (Leica Biosystems). Gr1 staining was performed using an ImmPRESS Goat anti-rat (Mouse absorbed IgG) Polymer Detection Kit (peroxidase) from Vector laboratories (MP-7444) according to the manufacturer’s instructions. Additional slides were subsequently stained for CD68 using a horseradish peroxidase-labeled polymer from Dako (K4003) according to the manufacturer’s instructions. Both Gr1 and CD68 staining were quantified by viewing slides on an Olympus BX41 light microscope. The cells stained for the antibody were counted manually in five high-power field sections at 20x magnification in a blinded manner.

### Measurement of Serum Alanine Aminotranferase and Aspartate Aminotransferase

ALT and AST levels were measured using a multichannel analyzer, Alfa Wassermann Vet Axcel, from the clinical diagnostics laboratory of VRL Maryland, LLC.

### Cell Preparation and Flow Cytometry

The specified liver tissues were collected into RPMI-1640 culture medium (Gibco). Liver tissues were passed through a 70-μm cell strainer (Fisher Scientific), and leukocyte fractions were isolated *via* Percoll (Cytiva) density gradient. After Percoll gradient centrifugation at 1000xg (25°C), without brake for 20 min, the upper layer, including cell debris, was carefully discarded. Leukocyte layer was washed and resuspended in 1X PBS (Gibco). Liver leukocytes were stained with the following antibodies for flow cytometry analysis: PE-conjugated anti-CD49b (BioLegend), APC-conjugated anti-CD49a (BD Biosciences), Brilliant Violet 605™-conjugated anti-NK-1.1 (BioLegend), Alexa Fluor^®^ 700-conjugated CD45 (BioLegend), PerCP-eFluor 710-conjugated anti-EOMES (eBioscience), PE/Dazzle™ 594-conjugated anti-T-bet (BioLegend), Brilliant Violet 510™-conjugated anti-CD11b (BioLegend), Brilliant Violet 711™-conjugated anit-CD107a (BioLegend), Brilliant Violet 650™-conjugated anti-CD27 (BioLegend), APC-conjugated anti-Perforin (BioLegend) and PE/Cyanine7-conjugated anti-Granzyme B (BioLegend). The lineage cocktail for mouse cells consisted of FITC-conjugated anti-CD3, CD5, CD19, Ly-6C, and CD11c (BioLegend). For ILC3, PE/Dazzle™ 594-conjuated anti-CD-127 (BioLegend), PE-Cyanine7-conjuated anti-NKp46 (eBioscience) and Alexa Fluor^®^ 647-conjuated anti-RORγt (BD Biosciences). Data were acquired using a BD FACSAria III Cytometer (BD Biosciences) at our Flow Cytometry & Cell Sorting Shared Resource (FCSR). Any samples with the viability of 60% or lower (as determined by staining with live dead marker Zombie NIR™, BioLegend) were excluded from all analyses. Fluorescence minus one (FMO) controls were used for analysis of cell surface staining, isotype controls were used for T-bet and Eomes transcription factor staining, and unstimulated controls (absence of PMA/ionomycin restimulation) were used for all intracellular cytokine staining (**Supplementary Figure 1A**).

### Cytokine Stimulation and Intracellular Cytokine Staining

Intracellular staining for the detection of cytokines was carried out from liver leukocytes. Approximately 1x10^6^ cells/ml RPMI supplemented with 10% FBS, 1% Penicillin Streptomycin, and 0.5% Gentamycin were cultured for 20 hours at 37°C in a cell culture flask. Recombinant mouse cytokine concentrations used were 10 ng/ml IL-12, 50 ng/ml IL-18, and 50 ng/ml IL-15 (R&D Systems). Cells were stimulated for 4 hours with Cell Activation Cocktail containing phorbol-12-myristate-13-acetate (PMA, 50ng/ml) and ionomycin (BioLegend) in the presence of 5 μg/ml brefeldin A (BioLegend). Following stimulation, the cells were stained with the following antibodies to detect cytokines: Brilliant Violet 650™-conjugated anti-IFN-γ (BD Biosciences) and Brilliant Violet 605™-conjugated anti-TNF-α (BioLegend). Cells were fixed (IC Fixation buffer, Invitrogen) and permeabilized (Permeabilization buffer, Invitrogen) according to manufacturer’s instructions.

### Real-Time PCR Array

Mouse liver specimens were stored in Allprotect Tissue Reagent (Qiagen). Total RNA was extracted using the RNeasy Mini kit with RNase-free DNase set (Qiagen). cDNA was synthesized utilizing RT² First Strand kit (Qiagen) followed by polymerase chain reaction (PCR) amplification and quantification using RT² Profiler™ PCR array for Mouse Cytokines & Chemokines (PAMM-150ZC-12, Qiagen). A total of 1.25µg RNA was pooled from at least three separate mice livers in an equal proportion to PCR profiler array. For the StepOnePlus (Applied Biosystems) thermocycler, the qPCR cycling conditions were 95°C for 10 minutes for 1 cycle, followed by 40 cycles of 95°C for 15 seconds and 60°C for 1 minute. Raw Ct values were analyzed to determine fold change and fold regulation using the GeneGlobe web tool (https://geneglobe.qiagen.com/us/). Non-IRI and IRI groups were tested separately for clustering analysis, considering IRI surgery-based changes in the Ct values for the housekeeping genes. Unsupervised hierarchical clustering for the 2^-ΔCt^ values was performed using the ClustVis (14) (https://biit.cs.ut.ee/clustvis) web tool to identify gene clusters specifically in non-IRI and IRI groups under two different diet regimens in *Rag1^−/−^* and Rag1-Tbet DKO mice and normalized using Actb and Hsp90ab1 as housekeeping genes. IRI-mediated genotype- and diet-specific fold regulation in gene expression was computed using respective ND (non-IRI) as a baseline and normalized using a housekeeping gene panel (Actb, Hsp90ab1, Gapdh) for *Rag1^−/−^* mice and automatically selected reference genes from the entire cataloged array (Nodal, IL-9, and Csf1) for Rag1-Tbet-DKO mice.

### Phasor-FLIM Imaging for Steatosis Calculation

Autofluorescence from 5 µm thick liver sections was imaged using the homebuilt DIVER (Deep Imaging *via* Enhanced Recovery) microscope (15, 16). The details of this microscope have been described elsewhere (16–19), and it is a homebuilt modified detector based on an upright configuration. DIVER is connected to a FastFLIM (ISS, Champaign, IL) acquisition card that calculates the fluorescence decay from each pixel of decay and transforms them to the phasor plot (20–22). Signals in the blue part of the fluorescence spectra were collected using a custom filter (400 – 500 nm) in combination with two BG39 filters that constitute the incoming and outgoing window of the filter. The phasor plot is calibrated using Rhodamine 110 in water with a mono-exponential lifetime of 4.0 ns.

Steatosis calculation is based on identifying lipid droplets and then plotting the sizes. The position of long lifetime species (LLS) in the phasor plot was selected using the red circle (19, 21, 23) (**Figure 2B**), and the FLIM images were colored accordingly. LLS is a signature that can only be found in lipid droplets. Very small LLS areas can be caused by a lower signal-to-noise ratio of the acquired fluorescence photons, increasing the spread of the phasor plot. A lower threshold value of connected pixels of size 40 pixels were used to eliminate this misidentification. The individual droplet size distribution from each sample were calculated, and the average size of the droplets were plotted to see changes between sample groups.

### Statistical Analysis

Mann–Whitney U test statistics and Multiple t-tests were performed using Prism Software (GraphPad, Inc. San Diego). All graphs show mean ± SEM unless stated otherwise. All p values presented were two-sided, and p<0.05 was considered statistically significant. The statistical difference between the samples in steatosis calculation using phasor-FLIM was calculated using student’s t-test and Origin software (OriginLab Corporation, Northampton, MA).

## Results

### HFD-IRI Mice Have Significant Increases in ILC1 Frequencies and Produce More IFN-γ and TNF-α When Compared to ND-IRI Mice

**Figure 1:**
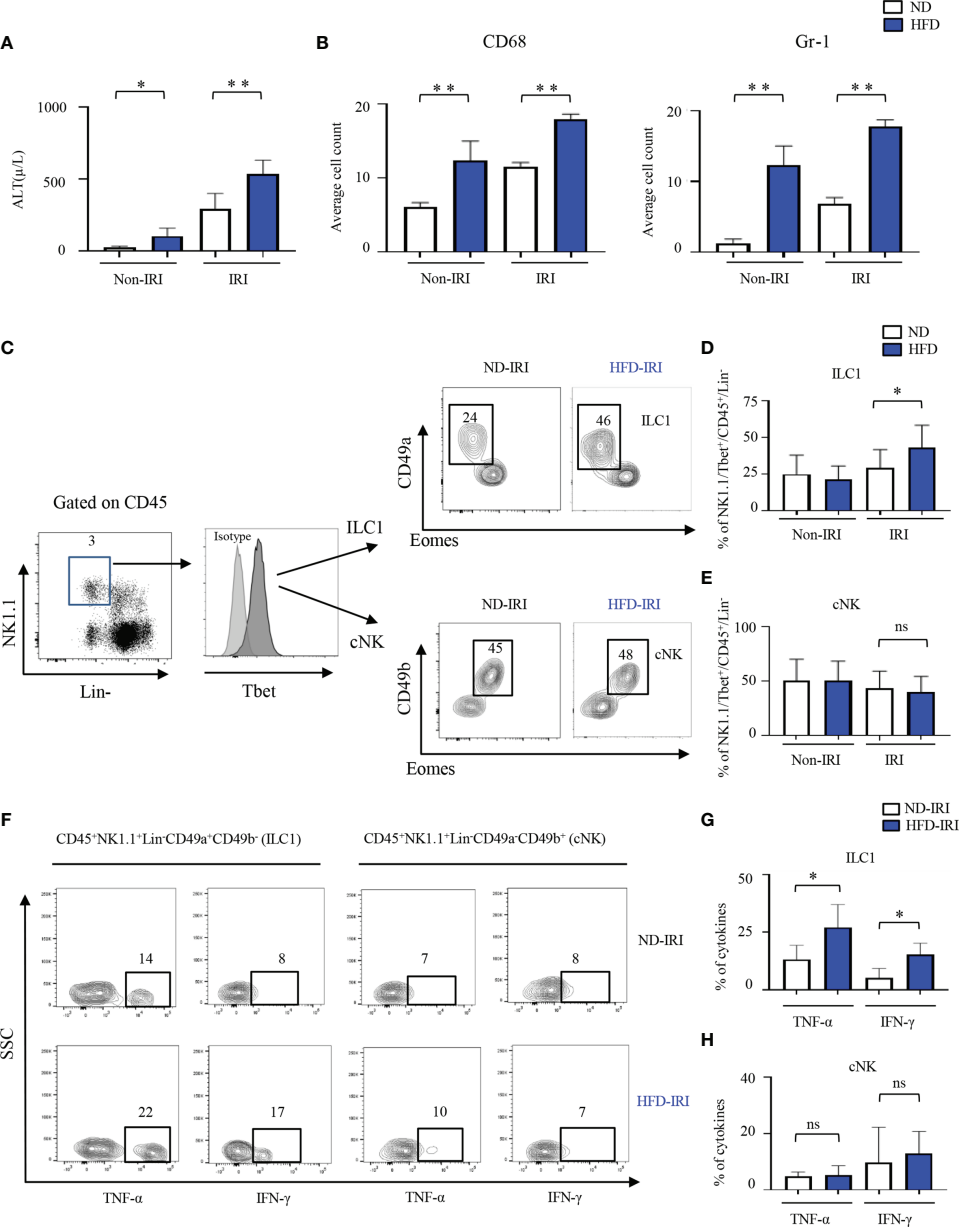
ILC1s are elevated in the liver of C57B/6 wild-type HFD-IRI mice and produce IFN-γ and TNF-α. C57B/6 wild-type mice were fed either standard chow (normal diet, ND) or a lard-based high fat diet (HFD). HFD was started at five-to-seven weeks of age and maintained for 12 weeks. All mice were 17-23 weeks of age at the time of experimentation. A 45-minute partial warm ischemia time was used for IRI experiments. All analysis was performed following 24 hours of reperfusion. **(A)** Serum alanine aminotransferase (ALT), the indicator of liver damage, was measured (n=3-7 mice). **(B)** Numbers of the infiltration of inflammatory cells (i.e., CD68^+^ and Gr1^+^) in the ND and HFD mice after IRI were quantified (n=9-10). **(C)** Gating strategy of ILC1s and cNKs from the hepatic lymphocytes: lineage- negative (Lin^-^) NK1.1^+^ cells expressing CD45 were identified as ILC1s and cNKs by expression of CD49a, CD49b, T-bet, and Eomes. Representative flow plots showing ILC1 and cNK subsets in ND-IRI and HFD-IRI. The percentages of ILC1s **(D)** and NKs **(E)** in ND and HFD mice after non-IRI or IRI (n=7-16 mice). **(F)** Representative flow plots showing intracellular cytokine production (IFN-γ^+^ and TNF-α^+^ cells) in ND-IRI and HFD-IRI. Fresh hepatic lymphocytes were stimulated with PMA/ionomycin for 4 hours in the presence of IL-12, IL-15, and IL-18. FACS plots gated on live, CD45^+^, Lin^-^, NK1.1^+^, CD49a^+^ and CD49b^-^ for ILC1s and CD45^+^, Lin^-^, NK1.1^+^, CD45a^+^ and CD45b^+^ for cNKs. The percentage of cytokines from ILC1 gated **(G)** and NK gated **(H)** cells in ND-IRI and HFD-IRI mice (n=5 mice per group). Significance was determined using Mann Whitney Test. *p<0.05; **p<0.01; ns, not significant.

C57BL/6 wild-type high fat diet (HFD) mice gained significantly more body weight (averaging 47.7g versus 26.8g at age 20 weeks; p<0.0001) than normal diet (ND) mice. In the first set of experiments, circulating alanine transaminase (ALT) activities were used as indicators of hepatocellular injury. The serum ALT levels of HFD mice were higher (p<0.05) than those of mice fed a ND (**Figure 1A**). Compared to normal diet IRI (ND-IRI) mice, the ALT levels of high-fat diet IRI (HFD-IRI) mice were significantly increased (p<0.01), as shown in **Figure 1A**. IRI-mediated hepatocellular inflammation was then determined using CD68 and Gr1 immunohistochemistry staining (**Supplementary Figure 1B**). The numbers of infiltrating CD68^+^ macrophages and Gr1^+^ neutrophils in HFD-IRI mice were significantly increased when compared to ND-IRI mice (**Figure 1B**), indicating that HFD mice exhibited exacerbated liver injury under IRI conditions.

Given the critical role of group 1 ILCs in regulating hepatic immune responses in liver inflammation, we then investigated the role of ILC1 and cNK cells in naïve non-IRI and IRI livers of HFD mice compared to ND mice *via* polychromatic flow cytometry (**Figure 1C**). We did not observe any significant difference in frequencies of ILC1s (defined as Lin^−^NK1.1^+^CD49a^+^CD49b^−^Tbet^+^Eomes^−^) or cNKs (defined as Lin^−^NK1.1^+^CD49a^−^CD49b^+^Tbet^+^Eomes^+^) in naïve non-IRI livers of HFD mice compared to ND mice (**Figures 1D, E**). However, HFD-IRI mice showed a significant increase in frequencies of ILC1s (**Figure 1D**) but not of cNKs (**Figure 1E**). In line with this, the absolute cell numbers of ILC1s were significantly increased in the HFD-IRI mice when compared to ND-IRI mice (**Supplementary Figure 2A**), while the absolute cell numbers of cNKs were not different (**Supplementary Figure 2B**).

Having demonstrated that ILC1s are a dominant innate cell population in the livers of HFD-IRI wild-type mice, we speculated that they are also a major source of proinflammatory IFN-γ and TNF-α. To validate this, we restimulated hepatic lymphocytes *ex vivo* with IL-12, IL-15, and IL-18 and found that ILC1s from HFD-IRI mice produced significantly more IFN-γ (p=0.01) and TNF-α (p=0.03) when compared to cells isolated from ND-IRI mice (**Figures 1F, G**), indicating that ILC1s play a potentially important role as proinflammatory effector cells in fatty liver IRI. We did not observe a significant difference in the cytokine production from cNKs (**Figures 1F, H**).

### HFD *Rag1^−/−^* and Rag1-Tbet DKO Mice Develop Comparable Levels of Hepatic Steatosis and Are a Novel Model for Studies of Group ILC1s

To assess whether ILC1s are truly key proinflammatory effector cells in hepatic IRI of fatty livers, *Rag1^−/−^* mice were utilized, which are known to lack T cells and B cells and have functional ILC and NK cell compartments. We further established a HFD murine model within these mice by subjecting them to HFD for 12-15 weeks (HFD *Rag1^−/−^*) and compared them to *Rag1^−/−^* on ND (ND *Rag1^−/−^*) for 12-15 weeks. Importantly, HFD *Rag1^−/−^* mice demonstrated significantly more weight gain than ND *Rag1^−/−^* mice at 15 weeks (33.30g vs. 23.69g, p=0.0002).

Moreover, to validate that ILC1s are true effector cells in hepatic IRI of fatty livers, Rag1-Tbet DKO mice were generated through crossbreeding of *Rag1^−/−^* mice with *Tbx21^−/−^* mice, which in addition to T and B cells also lack T-bet dependent ILC1s but still possess Eomes^+^ cNKs. A HFD murine model was again created by subjecting Rag1-Tbet DKO mice to HFD for 12-15 weeks (HFD Rag1-Tbet DKO) and compared to Rag1-Tbet DKO mice, which received ND (ND Rag1-Tbet DKO). Importantly, HFD Rag1-Tbet DKO also showed significantly more weight gain than ND Rag1-Tbet DKO at 15 weeks (30.53g vs. 23.45g, p=0.0015). There was no statistically significant difference in body weights between Rag1-Tbet DKO and *Rag1^−/−^* mice under both ND and HFD conditions (averaging 23.5g and 23.7g for ND groups, p=0.853; and averaging 30.53g and 32.36g for HFD groups, p=0.346, respectively; **Supplementary Figure 3A**), confirming that ILC1-deficient Rag1-Tbet DKO mice possess a comparable HFD-phenotype to *Rag1^−/−^* mice.

**Figure 2:**
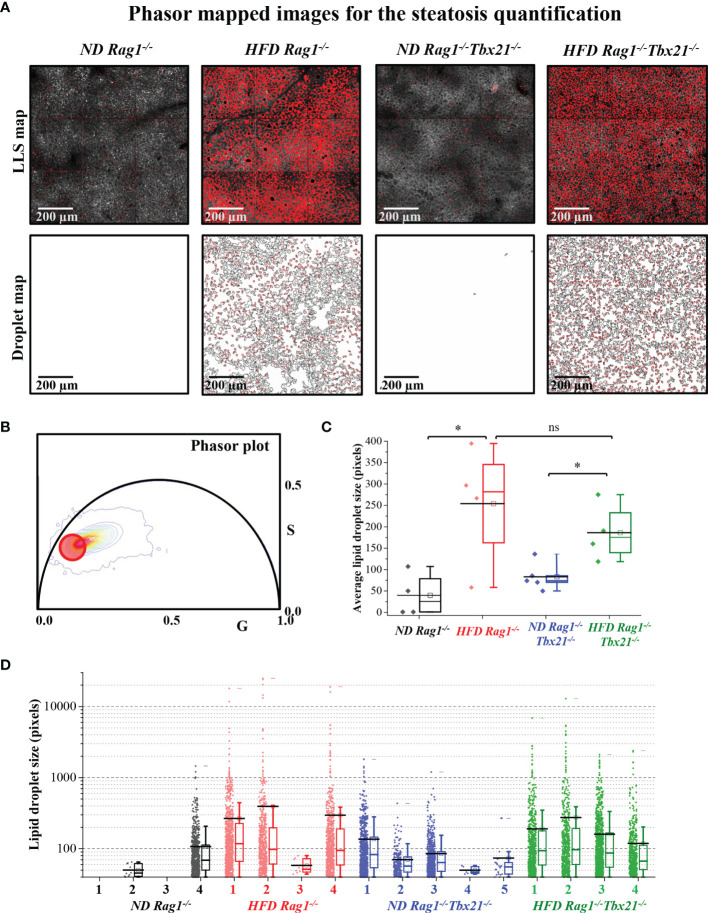
Steatosis quantification using long lifetime species of phasor-in Rag1^-/-^ and Rag1-Tbet DKO hepatic IRI mouse model. HFD was started at five-to-seven weeks of age and maintained for 12 weeks. All mice were 17-19 weeks at time of evaluation. **(A)** Fluorescence lifetime images were color mapped for long lifetime species (LLS) present in lipid droplets, and their size distribution was calculated using a custom ImageJ script (droplet map is shown in **Figure 2A**, bottom). **(B)** The LLS signal was selected based on the phasor-FLIM map (red circle). The calculated average lipid droplet sizes **(C)** and the individual droplet size distributions are plotted **(D)**. The excitation wavelengths used were 740 nm. Significance was determined using Mann Whitney Test. *p<0.05; ns, not significant. Each dot represents the value for a single mouse.

To corroborate this at a microscopic level, we next utilized Phasor-FLIM (Fluorescence lifetime imaging microscopy) to characterize hepatic steatosis in both *Rag1^−/−^* and Rag1-Tbet DKO mice undergoing IRI. For this, we subjected all mice to our 45-minute partial warm IRI model and evaluated snap-frozen liver tissue from the affected left lobe. Selection of the phasor signature of LLS (red circles, **Figure 2B**, as mentioned in Method 2.8) was then utilized to identify lipid droplets (**Figure 2A**) and calculate lipid droplet size (**Figure 2A**, bottom) based on a 40-pixel lower limit to avoid the appearance of this signature in low signal-to-noise (S/N) ratio areas of the image. As expected, our data demonstrated that HFD *Rag1^−/−^* mice IRI and HFD Rag1-Tbet mice DKO IRI mice both had significantly larger lipid droplets and distributions than their ND IRI counterparts. Moreover, lipid droplet morphologies were similar between ND *Rag1^−/−^* IRI and ND Rag1-Tbet DKO mice (p= not significant; ns) as well as between HFD *Rag1^−/−^* IRI and HFD Rag1-Tbet DKO mice (p=ns), confirming comparable levels of hepatic steatosis between both mouse strains under HFD-IRI conditions at a microscopic level (**Figures 2C, D**).

### HFD Rag1-Tbet DKO but Not *Rag1^−/−^* Mice Lack ILC1s and Are Protected From IRI in the Setting of Hepatic Steatosis

**Figure 3:**
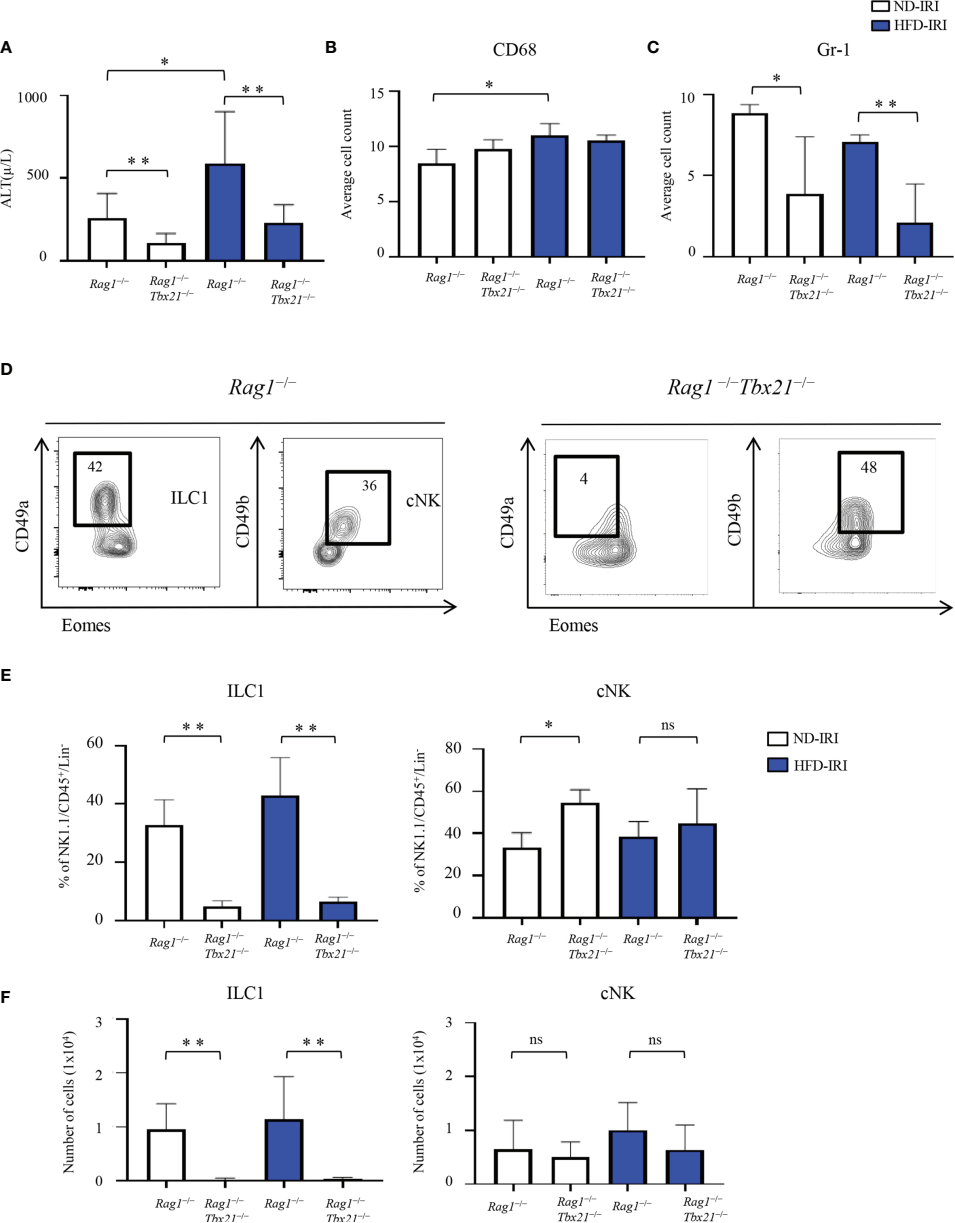
Absence of ILCs lead to increased liver protection to IRI. HFD was started at five-to-seven weeks of age and maintained for 12 weeks. All mice were 17-19 weeks at time of evaluation. A 45-minute partial warm ischemia time was used for IRI experiments. All analysis was performed following 24 hours of reperfusion. **(A)** Serum alanine aminotransferase (ALT) was measured in ND-IRI and HFD-IRI between *Rag1^−/−^* and Rag1-Tbet DKO mice (n=5-7). The numbers of CD68^+^ **(B)** and Gr1^+^ **(C)** in the ND-IRI and HFD-IRI mice were quantified (n=9-10). **(D)** Representative flow plots show that the frequencies of ILC1s were nearly absent in Rag1-Tbet DKO. Data in Figure **(D)** are representative of ND IRI *Rag1^−/−^* or Rag1-Tbet DKO mice. **(E)** Percentage **(F)** and the absolute number of ILC1s and cNKs in *Rag1* ^−/−^ or Rag1-Tbet DKO mice following ND-IRI and HFD-IRI (n=5-7). Significance was determined using Mann Whitney Test. *p<0.05; **p<0.01; ns; not significant.

Next, we evaluated the severity of IRI between both ND and HFD *Rag1^−/−^* and Rag1-Tbet DKO mice. HFD *Rag1^−/−^* mice had significantly higher ALT levels than ND *Rag1^−/−^* mice upon IRI (p=0.03, **Figure 3A**). Importantly, we did appreciate significantly higher ALT levels in HFD *Rag1^−/−^* mice than in HFD Rag1-Tbet DKO mice (p=0.002, **Figure 3A**). We also observed that HFD *Rag1^−/−^* mice had significantly higher AST levels than HFD Rag1-Tbet DKO mice (**Supplementary Figure 3C**). However, there was no statistical difference in ALT levels between ND and HFD Rag1-Tbet DKO mice, suggesting a relative protective IRI phenotype in HFD Rag1-Tbet DKO mice when compared to their HFD *Rag1^−/−^* counterparts.

We next utilized Gr1 and CD68 tissue staining to evaluate the influx of neutrophils and macrophages following IRI, respectively. Interestingly, the overall macrophage influx was similar between all mouse groups as indicated by CD68 immunostaining (**Figure 3B**). Critically, while there were no significant differences in frequencies of Gr1^+^ neutrophils as determined by Gr1 immunostaining between ND and HFD *Rag1^−/−^* mice or ND and Rag1-Tbet DKO mice, respectively, there was a significantly higher influx of neutrophils in *Rag1^−/−^* mice when compared to Rag1-Tbet DKO mice under both ND and HFD conditions, (**Figure 3C**, **Supplementary Figure 3B**). These findings further corroborate the increased protection against IRI afforded to Rag1-Tbet DKO mice, particularly, under hepatic steatosis conditions.

Given the greater degree of IRI demonstrated in HFD *Rag1^−/−^* mice, we next examined the existence of ILC1 and cNK cells in both ND and HFD *Rag1^−/−^* and Rag1-Tbet DKO mice following IRI (**Figure 3D**). Notably, the frequencies and absolute numbers of ILC1s were nearly absent in Rag1-Tbet DKO mice of both ND-IRI and HFD-IRI groups (**Figures 3E, F**), confirming a previous report (24). Interestingly, the frequencies of cNKs in Rag1-Tbet DKO mice were significantly higher compared to *Rag1^−/−^* mice (**Figure 3E**), whereas their absolute cell numbers were not significantly altered (**Figure 3F**). Finally, we studied T-bet-dependent ILC3 phenotype to characterize the ILC3 populations (defined as Lin^−^CD127^+^CD117^+^NK46^+^) present in our model. However, we did not observe any differences in the frequencies and absolute cell numbers of ILC3 between *Rag1^−/−^* and Rag1-Tbet DKO mice (**Supplementary Figures 4B, C**). Combined, these data confirm the presence of a substantial population of ILC1s in *Rag1^−/−^* mice, which may exacerbate IRI under HFD conditions.

### ILC1s Are Producers of IFN-γ in *Rag1^−/−^* Mice With Hepatic Steatosis Undergoing IRI

**Figure 4:**
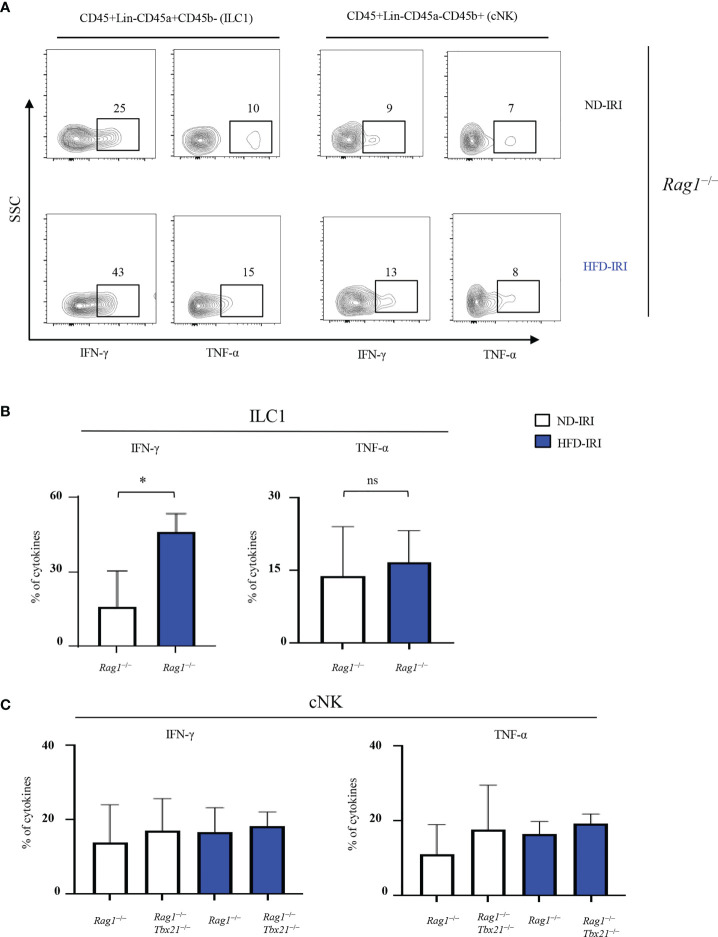
ILC1s produce higher IFN-γ in HFD-IRI. **(A)** Representative flow plots showing intracellular cytokine production (IFN-g^+^ and TNF-α^+^ cells) in ND-IRI and HFD-IRI *Rag1* ^−/−^ mice. FACS plots gated on live, CD45^+^, Lin^-^, NK1.1^+^, CD49a^+^ and CD49b^-^ for ILC1s and CD45^+^, Lin^-^, NK1.1^+^, CD45a^-^ and CD45b^+^ for cNKs. **(B)** Percentage of cytokines from ILC1 gated cells in ND-IRI and HFD-IRI *Rag1* ^−/−^ mice. **(C)** Percentage of cytokines from cNK gated cells in *Rag1* ^−/−^ and Rag1-Tbet DKO mice (n=3-5 mice per group). Significance was determined using Mann Whitney Test. *p<0.05; ns, not significant.

Thus, we then assessed the distinct contributions of ILC1s vs cNKs to IRI-induced inflammation in our HFD model by comparing their respective cytokine production in *Rag1^−/−^* and Rag1-Tbet DKO mice upon IRI. First, we determined the production of IFN-γ and TNF-α by ILC1s from *Rag1^−/−^* mice after IRI. Importantly, IFN-γ levels in ILC1s from HFD-IRI *Rag1^−/−^* mice were significantly higher than from ND-IRI *Rag1^−/−^* mice, while TNF-α levels were similar between both groups (**Figures 4A, B**). We also analyzed the IFN-γ and TNF-α production by cNKs from both *Rag1^−/−^* and Rag1-Tbet DKO mice after IRI and did not observe significant differences in cytokine production between groups (**Figure 4C**). Notably, we found that, in HFD-IRI *Rag1^−/−^* mice, ILC1s produced higher levels of IFN-γ than cNK cells indicating that hepatic ILC1s are a critical source of IFN-γ in HFD *Rag1^−/−^* mice, thereby contributing to exacerbated hepatic IRI of steatotic livers.

### *Rag1^−/−^* ND and HFD Mice Have Similar cNK Cells Maturation Characteristics, Whereas cNK Maturation and Perforin Frequencies Are Significantly Altered in Rag1-Tbet DKO Mice

**Figure 5:**
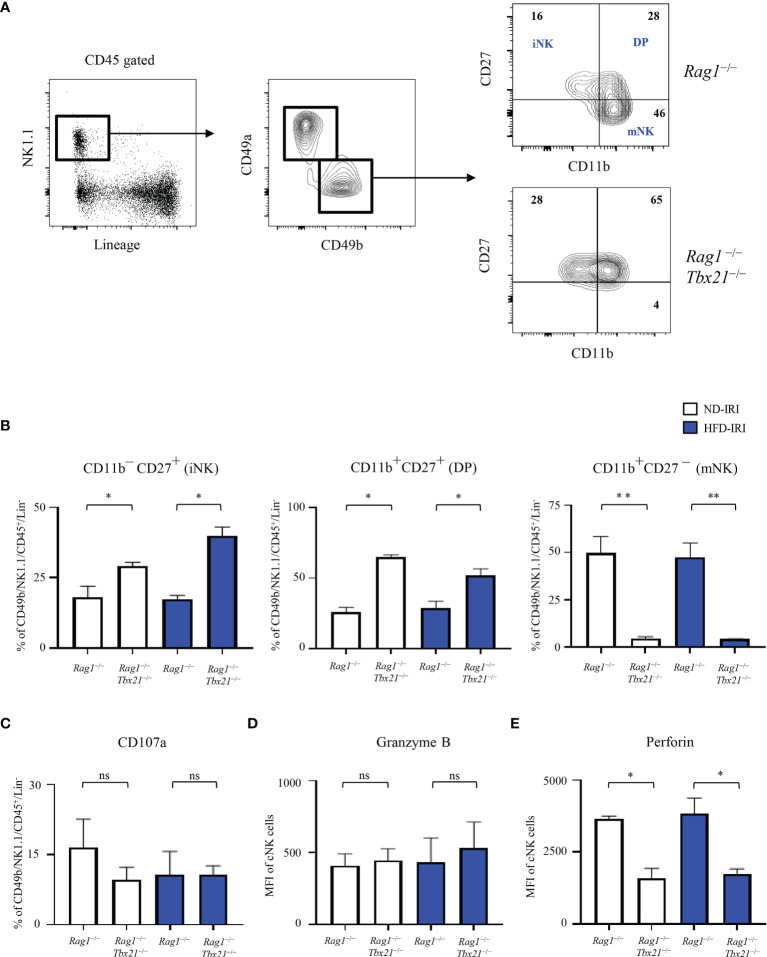
Lack of T-bet leads to a decrease in mNK cells and lower expression of perforin. **(A)** Gating strategy of cNKs from the hepatic lymphocytes: lineage- negative (Lin^-^) NK1.1^+^ cells expressing CD45 were identified as cNKs by expression of CD49a and CD49b. Immature NK (CD11b^−^ CD27^+^), Double Positive (CD11b^+ ^CD27^+^, DP) and mature NK (CD11b^+^CD27^−^) were identified by CD11b and CD27 expression, as shown. Representative flow plots **(A)** from ND-IRI *Rag1^−/−^* compared with ND-IRI Rag1-Tbet DKO mice and bar graph **(B)** showing NK cell developmental stages in IRI *Rag1^−/−^* compared with IRI Rag1-Tbet DKO mice. n=4 mice per group. **(C)** The frequency of CD107a staining of cNK cells from ND-IRI and HFD-IRI mice. Fresh hepatic lymphocytes were cultured for 4 h in the presence of anti-CD107a and Brefeldin **(A)** MFI of granzyme B **(D)** and perforin **(E)** staining in cNK cells from ND-IRI and HFD-IRI mice. n=4 mice per group. Significance was determined using Mann Whitney Test. *p<0.05. **p<0.01; ns, not significant.

Given that the T-bet transcription factor is required for cNK cell maturation (25) and our observation that the cNK frequencies and absolute cell numbers were similar in both *Rag1^−/−^* and Rag1-Tbet DKO mice, we next investigated the maturation phenotype and cytotoxic function present within our model contingent on HFD status and IRI. We utilized a CD11b and CD27 gating strategy to further differentiate NK1.1^+^/Lin^−^/CD49b^+^ cNK for immature CD11b^-^CD27^+^ (iNK), mature double positive CD11b^+^CD27^+^ (DP) cells, and terminally mature CD11b^+^CD27^-^ (mNK) cells (**Figure 5A**).

When comparing the ND and HFD *Rag1^−/−^* and Rag1-Tbet DKO, we demonstrated that these mice lack mNK cells in comparison to *Rag1^−/−^* mice, while they have significantly higher frequencies of iNK and DP positive cells. However, there were no significant differences in iNK, DP, or mNK cell populations between *Rag1^−/−^* ND and HFD groups or the Rag1-Tbet DKO ND and HFD groups, respectively (**Figure 5B**).

We then studied the expression of degranulation and cytotoxic markers in NK cells under IRI and HFD conditions in our model, given that T-bet has been shown to promote the transcription of genes including perforin and granzyme B to activate NK cell cytotoxicity (9). We found that there were no significant differences in degranulation, as indicated by CD107a staining, and there were no differences in intracellular granzyme B staining (**Figures 5C, D**). However, we did find a significantly lower expression of perforin by cNK cells in the livers of Rag1-Tbet DKO mice when compared to *Rag1^−/−^* mice (**Figure 5E**) under IRI conditions in both the ND and HFD model. Interestingly, there was no difference in perforin expression between ND and HFD *Rag1^−/−^* mice under IRI conditions.

### Rag1-Tbet DKO Mice Show Altered Expression of ILC1-Associated Genes Following IRI

**Figure 6:**
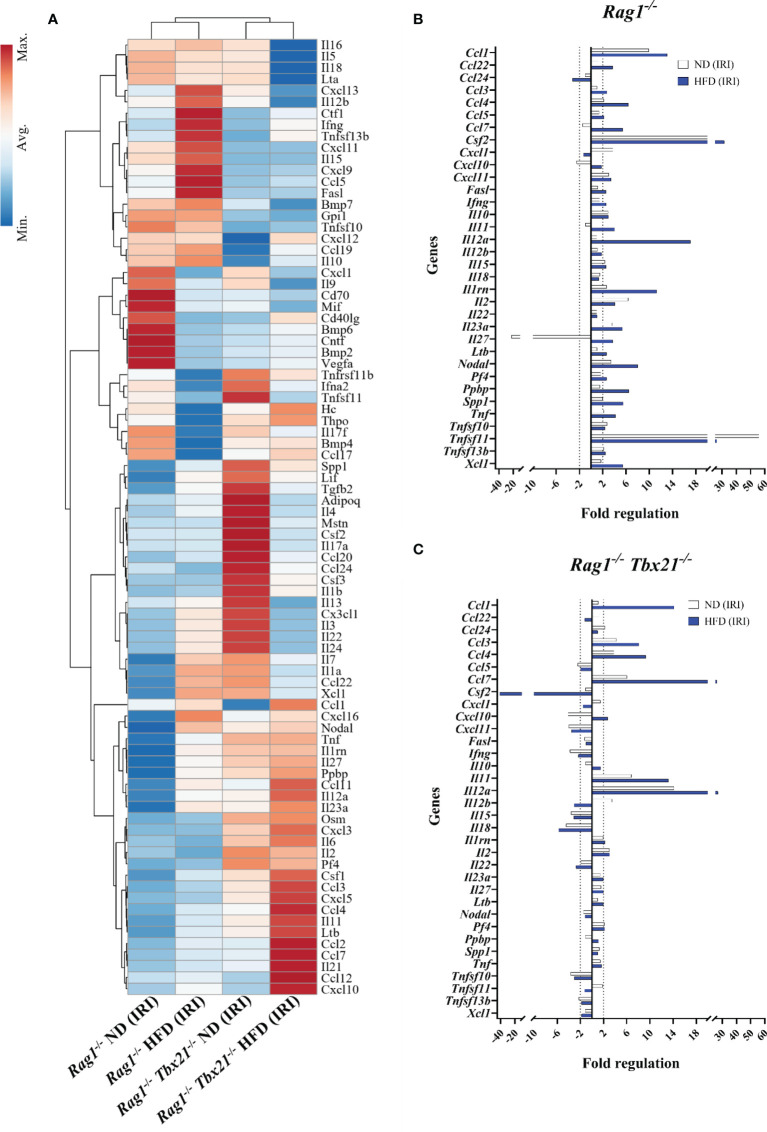
Cytokine and chemokine gene expression array for mice liver IRI. **(A)** Clustergram represents unsupervised hierarchical clustering analysis of RT^2^ qPCR profiler array for ND *Rag1^−/−^* HFD *Rag1^−/−^*, ND Rag1-Tbet DKO, and HFD Rag1-Tbet DKO pooled, post-IRI mice liver tissues. Comparative fold regulation analysis of post-IRI cytokines and chemokines with at least 2-fold or more up-and down-regulation between **(B)** ND and HFD *Rag1^−/−^* and **(C)** ND and HFD Rag1-Tbet DKO mice liver tissues relative to the respective ND non-IRI control. Dotted vertical lines mark up-and down-regulation of the gene expression. All data are represented as a gene expression of at least three pooled bulk mouse liver tissues.

Having established higher levels of ILC1-derived IFN-γ in HFD-IRI *Rag1^−/−^* mice, we validated ILC1 function within *Rag1^−/−^* mice and defined the relative contributions to immune signaling following IRI using the RT^2^ Profiler qPCR cytokine/chemokine array. To identify diet-specific gene clusters in *Rag1^−/−^* and Rag1-Tbet DKO mice, we performed the unsupervised hierarchical clustering approach for IRI (**Figure 6**) and non-IRI (**Supplementary Figure 5**) groups. We observed a pronounced ILC1-specific gene cluster in HFD *Rag1^−/−^* mice liver involving *IFNG*, tumor necrosis factor ligand superfamily member 10 (*TNFSF10*, also known as TNF-related apoptosis-inducing ligand *TRAIL*) and Fas ligand (*FasL*) and genes encoding ILC1-stimulatory cytokines such as *IL12B* and *IL15* in both non-IRI and IRI *Rag1^−/−^* mice but not in Rag1-Tbet DKO mice.

We then analyzed the fold-change differences between ND-IRI and HFD-IRI liver tissues in *Rag1^−/−^* (**Figure 6B**) and Rag1-Tbet DKO (**Figure 6C**) mice compared to respective non-IRI groups. Upon IRI, we observed higher expression of proinflammatory cytokines encoding genes including *IFNG*, *TNF*, and *CSF2* (GM-CSF) in both ND and HFD *Rag1^−/−^* mice (**Figure 6B**) compared to ND and HFD Rag1-Tbet DKO mice (**Figure 6C**), except IL-2, suggesting loss of Tbet-expressing ILC1s led to reduction in ILC1-derived effector cytokines. In a diet-independent manner, we also observed upregulation of the gene encoding *TRAIL* in *Rag1^−/−^* mice as opposed to in Rag1-Tbet DKO mice. ILC1 cells can produce IFN-γ under the stimulation of IL-12, IL-15, and IL-18 (26). In IRI liver tissues, we observed an upregulation of *IL12b*, *IL15*, and *IL18* genes in *Rag1^−/−^* mice while there was downregulation of the same genes in HFD Rag1-Tbet DKO mice (**Figure 6B**).

## Discussion

Recent studies have shown the intricate interplay between ILC1s and their environment in regulating immune-mediated liver disease through the secretion of IFN-γ. However, little is known about the phenotype and function of ILC1s in the setting of hepatic ischemia-reperfusion injury, specifically in liver transplantation using “marginal” allografts. In this study, we utilized a HFD murine model in wild-type and genetically modified mice to help investigate the role of ILC1 in a partial warm ischemia-reperfusion injury model.

We first analyzed the ILC1 and cNK cell frequencies, and associated proinflammatory cytokine secretion, in wild-type mice following IRI. Our flow cytometric analysis distinguished significantly greater enrichment of IFN-γ and TNF-α producing ILC1s, compared to cNK cells, in HFD mice following IRI. This is consistent with our previous work in human intestinal transplantation, demonstrating increased ILC1 populations in intestinal allografts 24 hours after reperfusion (27). Further studies in human samples have demonstrated that the proportion of ILC1s is increased in chronic hepatitis B patients compared to healthy controls, indicating a potential proinflammatory role of ILC1s (28) in liver diseases. This has also been alluded to using comparative studies of inflammation in both lung and kidney murine models. Specifically, the ILC1 population has been demonstrated to be higher in kidney specific IRI (29) and the overall ILC populations are higher in mice exposed to cigarette smoke than in control mice (30). Taken together, our initial data distinguishes the potential proinflammatory role of ILC1s in hepatic IRI under steatotic conditions.

While we hypothesized that ILC1s have a proinflammatory effect in our model, there have also been reports of a potential protective role of ILC1s in other models. Specifically, Nabekura et al. utilized a carbon tetrachloride (CCl4)-mediated liver injury model to show that activated ILC1s secrete IFN-γ, which subsequently promotes hepatocyte survival (11). This potentially alludes to the importance of ILC plasticity, as previously demonstrated (12). Cuff et al. demonstrated the plasticity of ILC1s showing a conversion of NK (defined as Lin-negative NK1.1^+^CD49a^−^CD49b^+^) cells into ILC1-like cells (CD200r1^+^ CD49a^+^) in mice with non-alcoholic fatty liver disease (31), thus indicating that cNKs could also differentiate into proinflammatory ILC1s in our HFD IRI model thereby exacerbating hepatocellular injury *via* IFN-γ upon IRI in the setting of hepatic steatosis, where the same degree of injury may not be appreciated in non-steatotic livers.

To further investigate and characterize the role of ILC1s in IRI, we utilized *Rag1^−/−^* mice that have substantial frequencies of cNK cells and ILC1s but lack T and B cells, in comparison to Rag1-Tbet DKO mice which additionally also lack T-bet-dependent ILC1s but retain Eomes-expressing cNK cells. We first demonstrated that HFD *Rag1^−/−^* mice have a more severe IRI than ND *Rag1^−/−^* and all Rag1-Tbet DKO mice by ALT levels and Gr1 (neutrophil) cell counts. This is an important finding, as it is well known that neutrophils contribute to IRI by inducing hepatic apoptosis and fibrosis (32, 33). Conversely, the number of CD68 positive cells (macrophages) was not significantly altered (**Figure 3B**) between HFD *Rag1^−/−^* and HFD Rag1-Tbet DKO mice after IRI. This is not surprising, as macrophages have a dual proinflammatory and anti-inflammatory role characterized by intrinsic polarization into either type 1 or type 2 macrophages (34–37).

We then noted that the frequencies of Eomes^+^ cNK-derived IFN-γ levels in *Rag1^−/−^* mice were comparable with Rag1-Tbet DKO mice. While the cytotoxic role of IFN-γ secreting liver resident cNK cells has been previously described (38, 39), and it is known that CD39 ablation is correlated with reduced IFN-γ-dependent responses by NK cells (13), our results did not correlate to clinical phenotype noted in our *Rag1^−/−^* HFD IRI model. Specifically, it is notable that there were no significant differences in absolute cell numbers of cNK cells or in the iNK, DP, and mNK cell populations between *Rag1^−/−^* ND and HFD groups, despite more severe IRI in the HFD *Rag1^−/−^* than in the ND *Rag1^−/−^* mice. This further supports that ILC1s have a contributory, proinflammatory role in IRI in the setting of hepatic steatosis. However, it is known that T-bet is a key transcription factor which results in the differentiation of iNK cells to mNK cells (9, 25), and it has been demonstrated that perforin-deficient mice develop less fibrosis in a model of non-alcoholic fatty liver disease, suggesting that reduction in cNK cell cytotoxicity is protective in the steatotic liver (31). To this regard, it remains possible that cNK cells and the apparent differences in perforin between the *Rag1^−/−^* and Rag1-Tbet DKO mice may contribute to the cytotoxicity of IRI in our model, as it has previously been demonstrated in a model of renal IRI (40). Moreover, given the emerging evidence supporting plasticity between cNK cells and ILC1, these cell types could be an intermediate phenotype in the differentiation from cNK to ILC1. More investigation is warranted to fully address whether this plasticity is present under HFD and IRI conditions.

We then evaluated the ILC1 frequency and associated ILC1 dependent IFN-γ production, which demonstrated significantly higher levels of ILC1s in *Rag1^−/−^* mice, as compared to the cNKs. This ultimately indicates that ILC1 is a major source of IFN-γ. It has been described that IFN-γ-deficient mice are significantly protected against liver injury and hepatic fibrosis in a model of non-alcoholic steatohepatitis (41), and it has further been demonstrated that anti-IFN-γ treatment results in lower ALT levels and hepatocellular injury following 48 hours of reperfusion in a model of 60-minute partial warm ischemia (42). However, the exact role of IFN-γ as secreted by ILC1 remains unknown. While our results suggest a proinflammatory role, additional investigations are warranted to truly evaluate the proinflammatory function of IFN-γ from ILC1s, for instance, through the use of a *Rag1^-/-^ IFN-γ^-/-^* deficient HFD mouse model.

We finally correlated this finding using our qPCR array to demonstrate the dynamic interplay of cytokines and chemokines in hepatic IRI. This highlighted the ILC1-specific elevated expression of TRAIL and CSF2 in *Rag1^−/−^* mice (**Figure 6B**) compared to Rag1-Tbet DKO mice (**Figure 6C**) independent of the diet regimens. Interestingly, our data also shows upregulation of FasL only in HFD *Rag1^−/−^* IRI but not in ND *Rag1^−/−^* IRI. FasL and TRAIL co-expression is more cytotoxic in hepatic ILC1 than the intestinal ILC1 (24). At the same time, independent studies in both mouse liver and kidney show that antibody-mediated inhibition of FasL is protective from liver failure (43) and ischemic acute kidney injury (44), respectively. Future investigation will be needed to identify the influence of hepatic steatosis on FasL-dependent immunomodulation and the exacerbation of liver injury.

Collectively, our findings indicate that hepatic ILC1s are, at least in part, an innate inflammatory effector subset, particularly in steatotic livers. Our findings provide deeper insights into the mechanisms of ILC1 and cNK cell function in IRI as well as identify further areas of interest in cNK cell to ILC1 plasticity and IFN-γ production in the setting of IRI and allow for additional translational studies in the use of “marginal allografts” in liver transplantation.

## Data Availability Statement

The original contributions presented in the study are publicly available. This data can be found in NCBI's Gene Expression Omnibus; GEO accession number: GSE205510.

## Ethics Statement

The animal study was reviewed and approved by The Georgetown University Institutional Animal Care and Use Committee.

## Author Contributions

AK and SR designed the study and secured funding. JK, JL, DP, SR, KL, YC, AD, and WC carried out experiments. JK, JL, DP, SR, and AK acquired data and wrote the manuscript. JK, JL, DP, BF, AD, SR, and AK analyzed data. AK, SR, WC, BK, DK, KO, KK, SR, and TF provided a critical review of the manuscript. All authors read and approved the manuscript.

## Funding

Funding was provided by NIH R21AI130800 (AK, SR) as well as by the Children’s Rare Disease Organization Inc. (CRDO) (AK, JK, KK).

## Author Disclaimer

The views expressed in this manuscript reflect the results of research conducted by the authors and do not necessarily reflect the official policy or position of the Department of the Navy, Department of Defense, or the United States Government.

JL is a military service member. This work was prepared as part of official duties. Title 17, USC, Section 105 provides that Copyright protection under this title is not available for any work of the U.S. Government and defines a U.S. Government work as a work prepared by a military service member or employee of the U.S. Government as part of that person’s official duties.

## Conflict of Interest

The authors declare that the research was conducted in the absence of any commercial or financial relationships that could be construed as a potential conflict of interest.

## Publisher’s Note

All claims expressed in this article are solely those of the authors and do not necessarily represent those of their affiliated organizations, or those of the publisher, the editors and the reviewers. Any product that may be evaluated in this article, or claim that may be made by its manufacturer, is not guaranteed or endorsed by the publisher.
