# Supplementary material for: Type 1 Innate Lymphoid Cells Are Proinflammatory Effector Cells in Ischemia-Reperfusion Injury of Steatotic Livers
Source: Front Immunol. 2022 Jun 27;13:899525. doi: 10.3389/fimmu.2022.899525 (PMC9272906; doi:10.3389/fimmu.2022.899525)
Supplement: Supplementary file 1 [file DataSheet_1.docx]

Type 1 innate lymphoid cells are proinflammatory effector cells in ischemia-reperfusion injury of steatotic livers

**Jiman Kang^1,2^, Jedson Liggett^1,3^, Digvijay Patil^1^, Suman Ranjit^2^, Katrina Loh^1^, Anju Duttargi^4^, Yuki Cui^1^, Kesha Oza^1^, Brett Frank^1^, DongHyang Kwon^5^, Bhaskar Kallakury^5^, Simon C Robson^6^, Thomas M Fishbein^1^, Wanxing Cui^1,2^, Khalid Khan^1^, Alexander Kroemer^1*^**

^1^ MedStar Georgetown Transplant Institute, MedStar Georgetown University Hospital and the Center for Translational Transplant Medicine, Georgetown University Medical Center, Washington, District of Columbia USA.

^2^ Department of Biochemistry and Molecular & Cellular Biology, Georgetown University, Washington, District of Columbia USA.

^3^ Naval Medical Center Portsmouth, Portsmouth, Virginia, USA.

^4^ Department of Oncology, Lombardi Comprehensive Cancer Center, Georgetown University Medical Center, Washington, District of Columbia, USA.

^5^ Department of Pathology, MedStar Georgetown University Hospital, Washington, District of Columbia, USA

^6^ Departments of Anesthesiology and Medicine, Beth Israel Deaconess Medical Center, Harvard Medical School, Boston, Massachusetts, USA.

## **Supplementary Data**

**Supplementary Figure 1**

(A) Representative FMO, isotype, and negative control. Fluorescence minus one (FMO) control was performed for all fluorescence and used for the gating setting. Isotype controls for the Tbet and Eomes were used to determine if the staining was specific. Cells incubated without PMA/ionomycin were used for negative control. (B) Representative images from immunohistochemistry of the CD68 (top) and Gr1 (bottom) staining (magnification, 20X) were performed on liver sections of ND, ND-IRI, HFD, and HFD-IRI. The inset shows higher magnification (40X). Bar = 100 μm.

**Supplementary Figure 2**

The number of ILC1s (A) and cNKs (B) in ND-IRI and HFD-IRI C57B/6 wild-type mice (n=5-7). Significance was determined using Mann Whitney Test. ns: not significant.

**Supplementary Figure 3**

(A) Body weight gain in *Rag1*^−/−^ and Rag1-Tbet DKO mice. Cumulative body weight change in ND or HFD-fed mice was measured weekly. Data were represented as mean ± SEM (n=7-29). Significance was determined using Multiple t-tests. The error bars represent the SEM. (B) Representative images from immunohistochemistry of the CD68 and Gr1 staining (magnification, 20X) were performed on ND-IRI and HFD-IRI mice liver sections. The inset shows higher magnification (40X). Bar = 100 μm. (C) Serum aspartate aminotransferase alanine (AST) was measured in ND-IRI and HFD-IRI between *Rag1^−/−^* and Rag1-Tbet DKO mice (n=5-7).

**Supplementary Figure 4**

ILC3 phenotype in *Rag1*^−/−^ and Rag1-Tbet DKO mice. (A) Representative flow plots showing ILC3 subsets in ND-IRI and HFD-IRI. The percentages (B) and numbers (C) of ILC3s in ND-IRI and HFD-IRI mice (n = 5–7) Significance was determined using Mann Whitney Test. ns: not significant.

**Supplementary Figure 5**

Cytokine and chemokine gene expression array for non-IRI mice liver tissue. Clustergram represents unsupervised hierarchical clustering analysis of RT^2^ qPCR profiler array for ND  *Rag1^−/−^*, HFD *Rag1^−/−^*, ND Rag1-Tbet DKO, and HFD Rag1-Tbet DKO for at least three pooled mice liver tissues. Data normalized to ND non-IRI as a baseline.


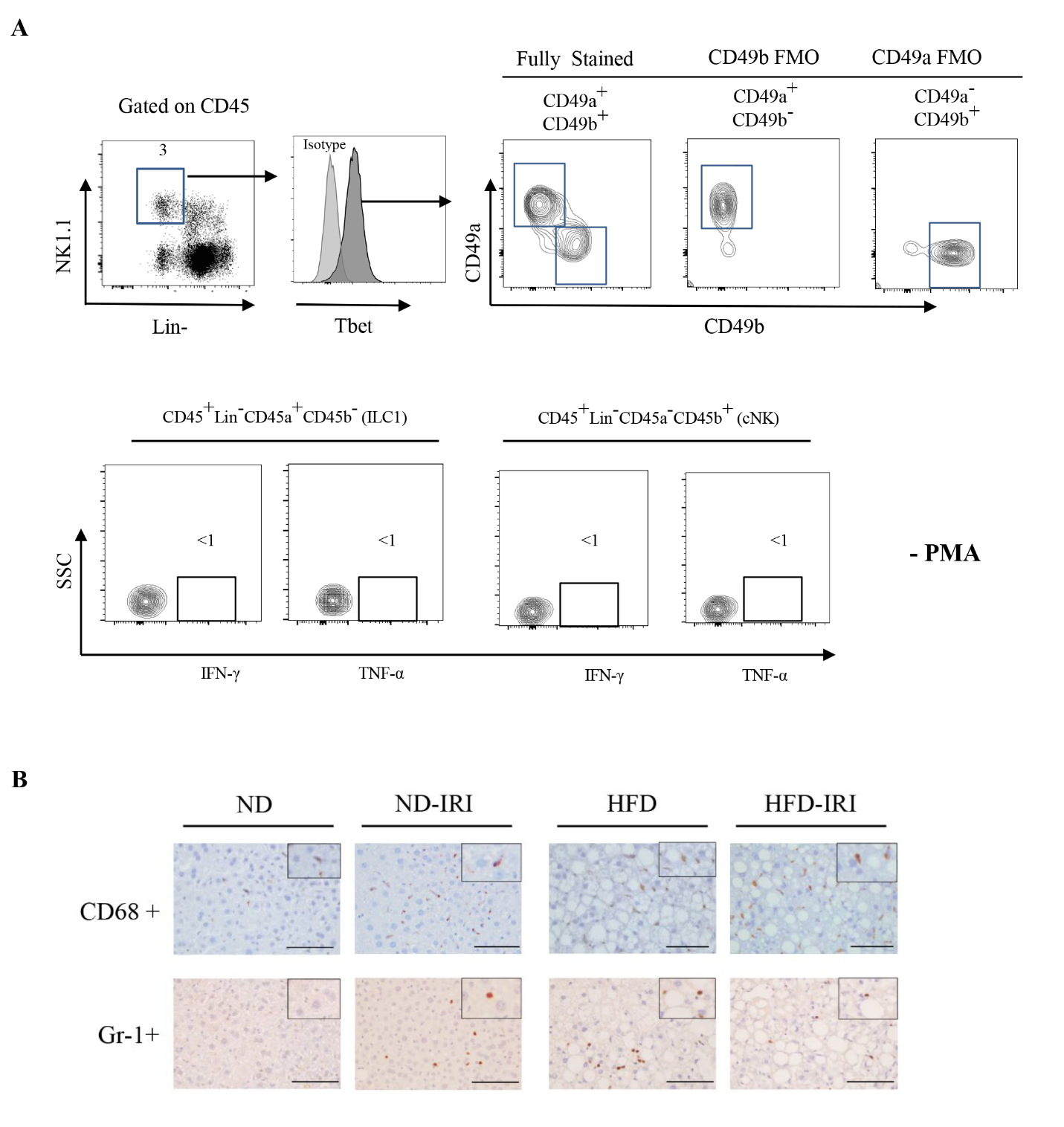
**Supplementary Figure 1**


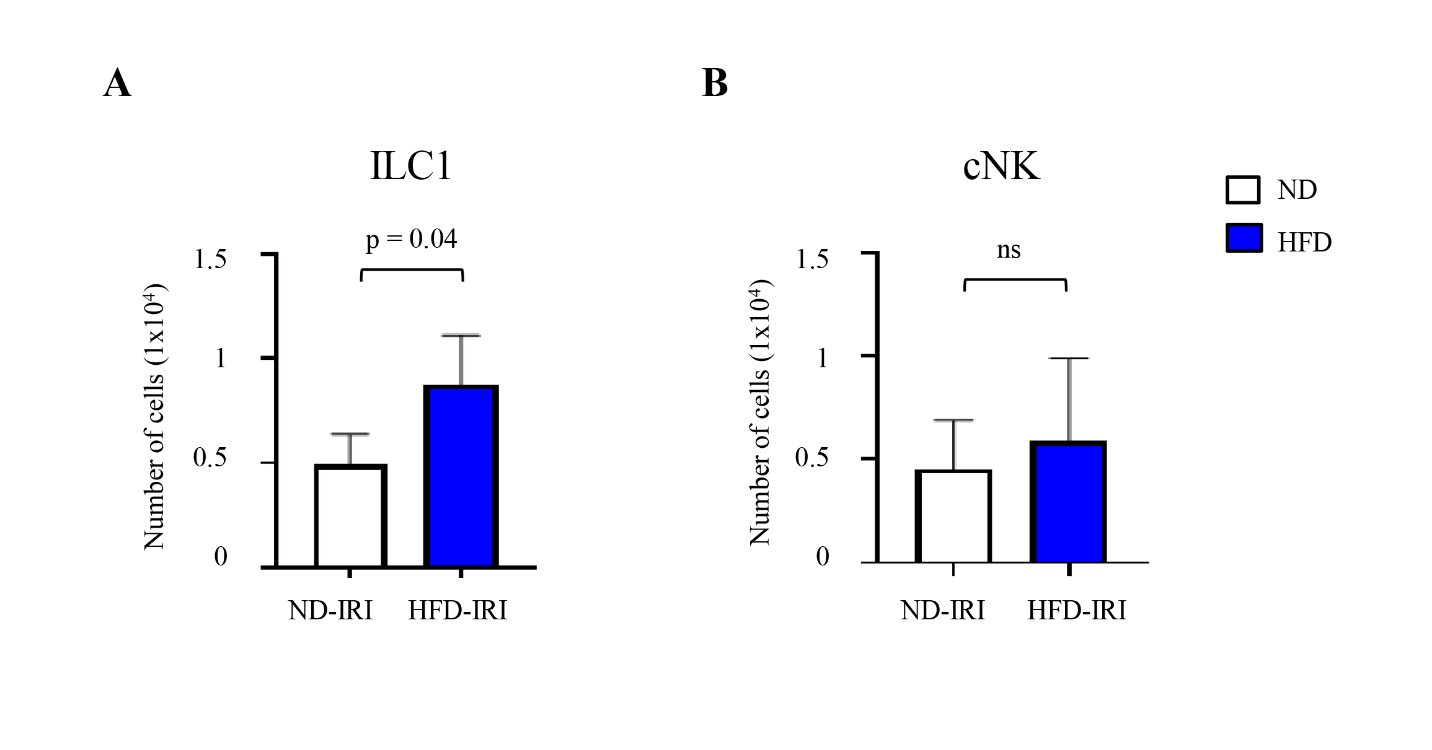
**Supplementary Figure 2**


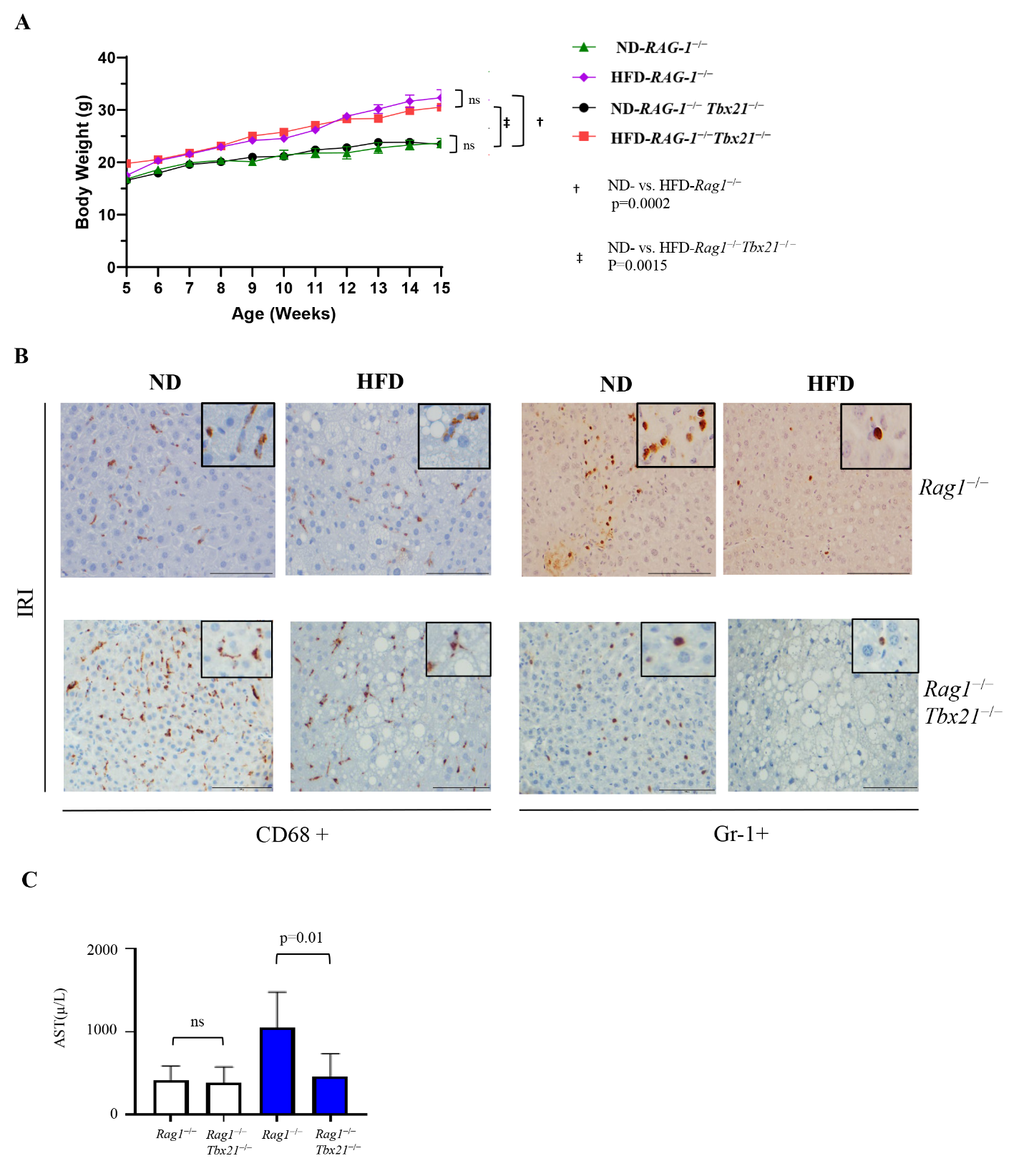
**Supplementary Figure 3**

**Supplementary Figure 4**

**
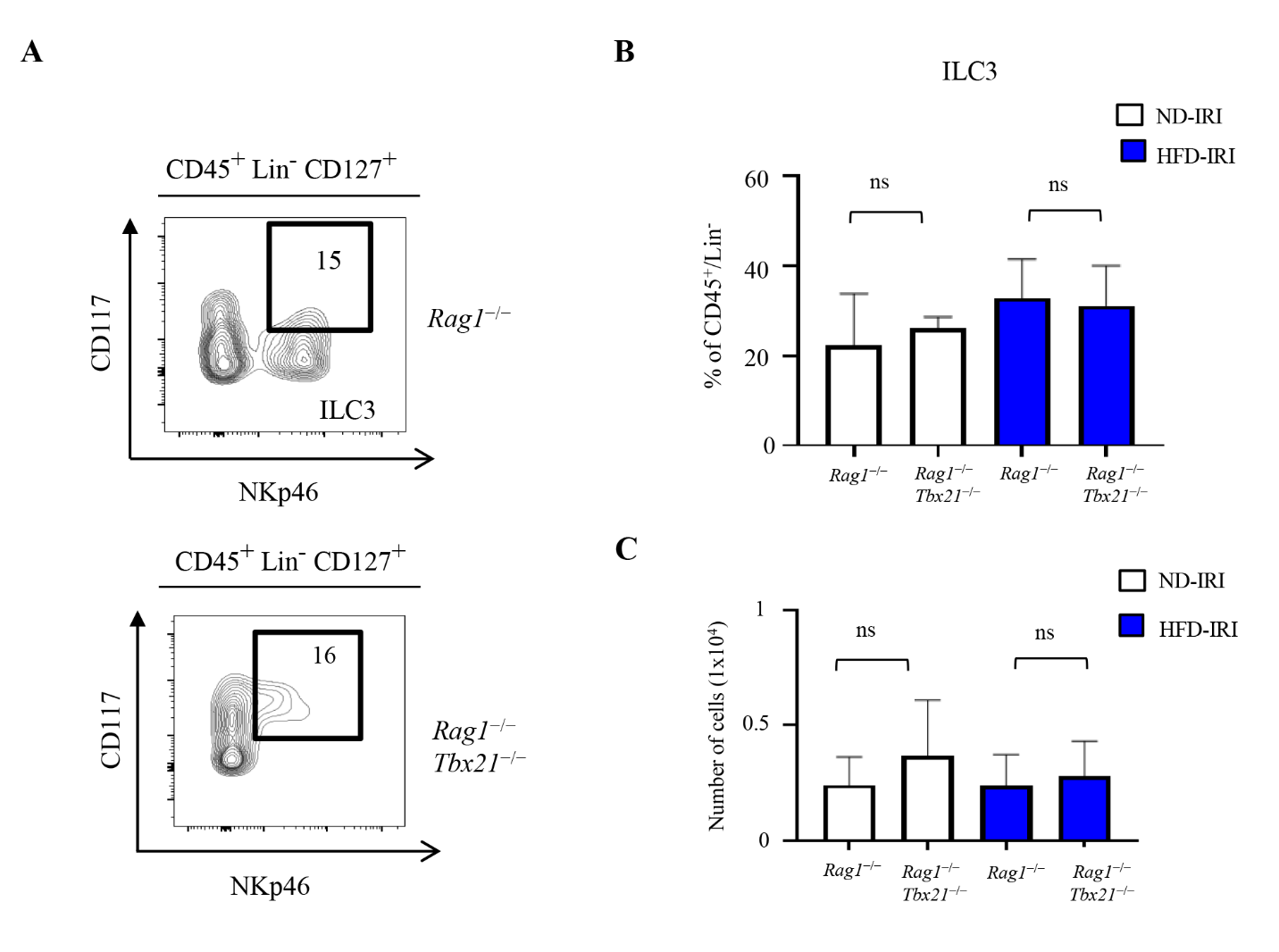
**


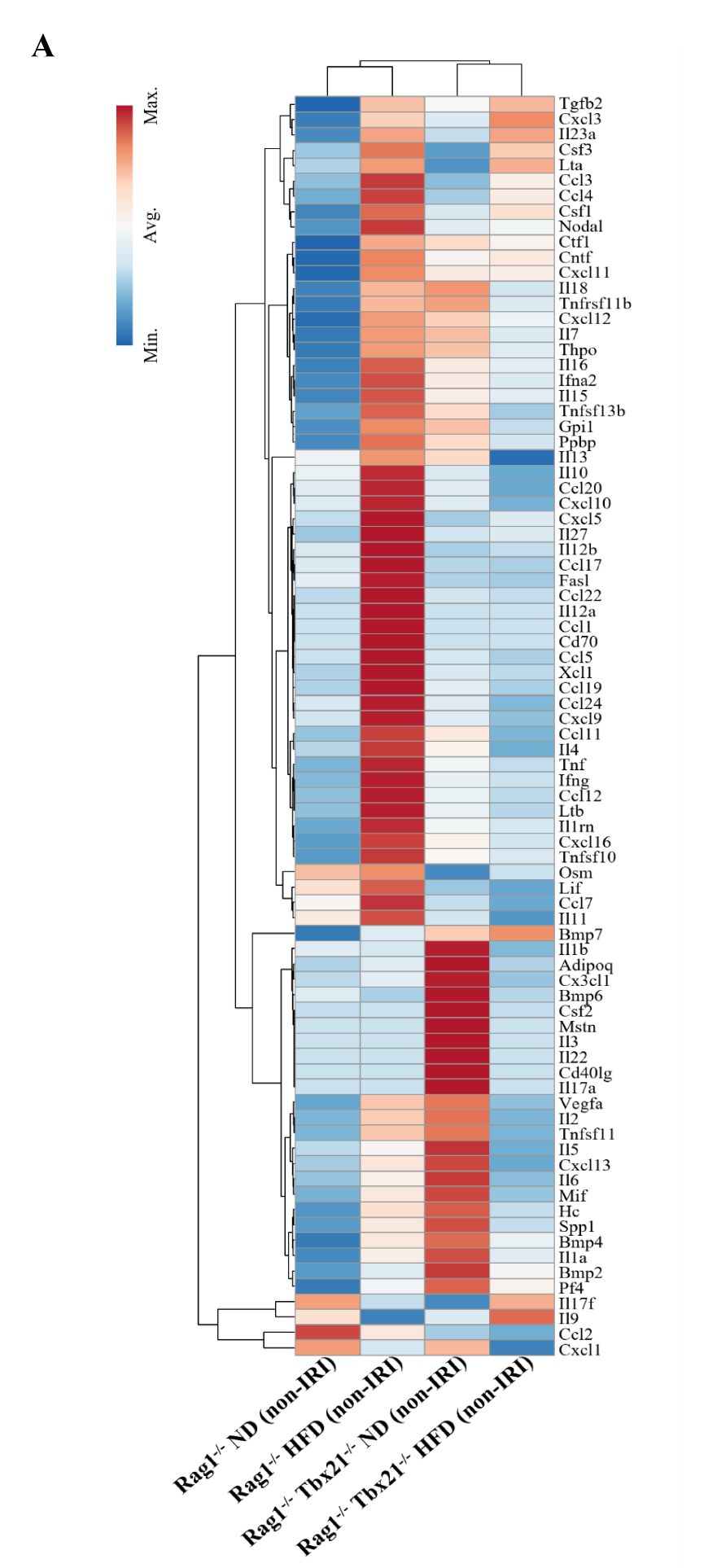
**Supplementary Figure 5**
